# The Emerging Role of Extracellular Vesicle-Derived lncRNAs and circRNAs in Tumor and Mesenchymal Stem Cells: The Biological Functions and Potential for Clinical Application

**DOI:** 10.3390/cancers17132186

**Published:** 2025-06-28

**Authors:** Ya-Wen Luo, Chen-Guang Liu, Jane Allyn Kirby, Chen Chu, Dan Zang, Jun Chen

**Affiliations:** 1 Department of Oncology, The Second Hospital of Dalian Medical University, Dalian 116021, China; yawen_luo@126.com (Y.-W.L.); 201972551@yangtzeu.edu.cn (C.-G.L.); 2 Department of Pediatric Oncology, Dana-Farber Cancer Institute and Department of Pediatrics, Harvard Medical School, Boston, MA 02215, USA; jane_kirby@dfci.harvard.edu; 3 Department of Cancer Biology, Dana-Farber Cancer Institute and Department of Genetics, Blavatnik Institute, Harvard Medical School, Boston, MA 02215, USA; chen_chu@dfci.harvard.edu

**Keywords:** mesenchymal stem cells, tumor, extracellular vesicles, lncRNAs, circRNAs

## Abstract

Extracellular vesicles (EVs) are released by nearly all types of cells, and they communicate between cells by transporting bioactive molecules, including proteins, DNA, RNA, and lipids. In recent years, RNA carried by EVs, particularly the long-chain non-coding RNA (lncRNA) and circular RNA (circRNA) subtypes, has garnered interest with respect to their role in controlling tumor progression. Among them, there are increasing reports that mesenchymal stem cell-derived extracellular vesicles (MSC-EVs) maintain a recently discovered function as transporters of lncRNAs and circRNAs. However, detailed molecular functions of lncRNAs and circRNAs contained in MSC-EVs are not presently summarized, and the efficacy of MSC-EVs as molecular carriers requires further elucidation. This review summarizes the biological characteristics of EVs and the common mechanisms of lncRNAs and circRNAs contained within them. The “double-edged sword” effect and related molecular mechanism of EV-derived lncRNAs (EV-lncRNAs) and circRNAs (EV-circRNAs) between differing tumor types and MSCs are highlighted. The potential of MSC-EVs in the field of tumor diagnosis and treatment is discussed to suggest new directions for the strategy of engineering MSC-EVs as anti-tumor drug carriers.

## Simple Summary

Extracellular vesicles (EVs) play a crucial role in intercellular communication between cancer cells and mesenchymal stem cells (MSCs). Long non-coding RNAs (lncRNAs) and circular RNAs (circRNAs) are involved in tumor regulation. In this review paper, we discuss the double-edged sword roles and the underlying mechanisms of EV-derived lncRNAs and circRNAs in different cancer types and MSCs. We describe in detail the biological characteristics of EVs, the mechanistic commonality of lncRNAs and circRNAs, and the value of EVs as clinical biomarkers. We also highlight the diagnostic, prognostic, and therapeutic potential of EVs and EV-derived lncRNAs and circRNAs.

## 1. Introduction

Mesenchymal stem cells (MSCs) exist in various tissues and possess capabilities for multi-directional differentiation [1,2]. Friedenstein and colleagues were the first to isolate and define MSCs from bone marrow, characterized by their adherence, high replicative capacity, and multipotent stem cell nature. MSCs exhibit abilities to self-renew and differentiate into diverse cell types, including mesodermal cells such as osteoblasts, chondrocytes, adipocytes, and hematopoietic matrix, making them among the most extensively studied stem cell populations [3,4,5]. MSCs can regulate the immune system by interacting with immune cells in multiple ways [6]. Given their versatility, self-renewal capacity, and low immunogenicity, MSCs have emerged as therapeutic targets for various diseases, including cancer. MSCs can play an important role by secreting extracellular vesicles (EVs), which have undeniable implications for novel therapeutic strategies [7,8,9].

EVs are cell-secreted, lipid-bilayer-delimited particles that lack replicative capacity [10]. EVs comprise a diverse assortment of vesicles generated via intracellular bodies or plasma membrane shedding. This classification includes exosomes, measuring 30 to 150 nm, medium-sized microvesicles (MVs) ranging from 100 to 1000 nm, and apoptotic bodies spanning 100 to 5000 nm [11,12]. Exosomes, originating from the endolysosome pathway, are encapsulated within multivesicular bodies (MVBs). Following fusion with the plasma membrane, exosomes are released into the extracellular space. MVs form membrane protrusions, eventually form bulges, and are finally separated. Apoptotic bodies emerge from blebs on the membrane of apoptotic cells [13,14,15]. Various types of cells release EVs under both physiological and pathological conditions [16]. And EVs are ubiquitously present in several body fluid types, including urine, serum, plasma, lymph, and cerebrospinal fluid [17,18]. EVs play a pivotal role in facilitating cell communication between proximate or distant target cells, mediating physiological effects such as immune regulation, intercellular signaling, and tissue repair. Simultaneously, they contribute to pathological processes such as cancer metastasis and pathogen transmission [19,20,21,22,23,24,25].

EVs can function as carriers to deliver and transport a variety of complex molecules, including proteins, nucleic acids, lipids, etc. Proteins associated with membrane transport, MVB-producing proteins, and tetraspanins are the most ubiquitous type of cargo carried by EVs [21,26,27]. Nucleic acids present in EVs exhibit considerable diversity, comprising microRNA (miRNA), long non-coding RNAs (lncRNAs), circular RNAs (circRNAs), transfer RNA (tRNA), small nuclear RNA, small nucleolar RNA (snoRNA), and mRNA [28,29,30]. RNA is divided into coding RNA and non-coding RNA (ncRNA). The coding RNA mainly refers to messenger RNA (mRNA), while there are many types of ncRNAs [31]. ncRNAs account for approximately 98% of the transcripts in the human genome. These ncRNAs are usually expressed in a tissue-specific manner and are associated with various biological functions [32]. It has been confirmed that ncRNAs can regulate the progression of tumors through various pathways, including genetic pathways such as mutations in the ncRNA genes themselves and abnormal changes in enzymes involved in the ncRNA pathway, as well as regulatory pathways such as epigenetics, transcription, or post-transcriptional regulation through abnormal expression of ncRNAs [33]. Lipid components within EVs include sphingomyelin, phosphatidylserin, phosphatidylinositol, phosphatidic acid, ceramide, and cholesterol [34]. Initially regarded as cellular waste, understanding of EV function has evolved beyond this nascent perception with the advent of advanced research methods and molecular detection technologies [35]. Subsequent investigations have revealed that EVs facilitate the targeted transport of non-coding RNAs (ncRNAs) to specific cells, thereby instigating precise regulatory functions [36,37]. Numerous studies demonstrate that the uptake of functional molecules carried by these EVs, particularly lncRNAs and circRNAs, exerts a regulatory influence over myriad physiological and pathological processes at the epigenetic, transcriptional, and post-transcriptional levels [36,38]. These processes include modulation of tumor growth, metastasis, angiogenesis, and drug resistance in tumor cells [17].

In recent years, numerous scientific publications have underscored the intricate relationship between EVs derived from MSCs (MSC-EVs) and the biological behavior of tumors, positioning them as a promising therapeutic strategy for a diverse array of diseases. This review provides an overview of the biological characteristics of EVs, the common mechanisms of lncRNAs and circRNAs, and conclusively encapsulates the distinctive regulatory network that exists between MSCs and tumors. Future anti-tumor therapeutic strategies targeting EV-derived lncRNAs (EV-lncRNAs) and circRNAs (EV-circRNAs), along with the clinical application potential of engineered MSC-EVs, are concurrently discussed in this review.

## 2. Biological Characteristics of EVs

### 2.1. Biogenesis of EVs

**Figure 1:**
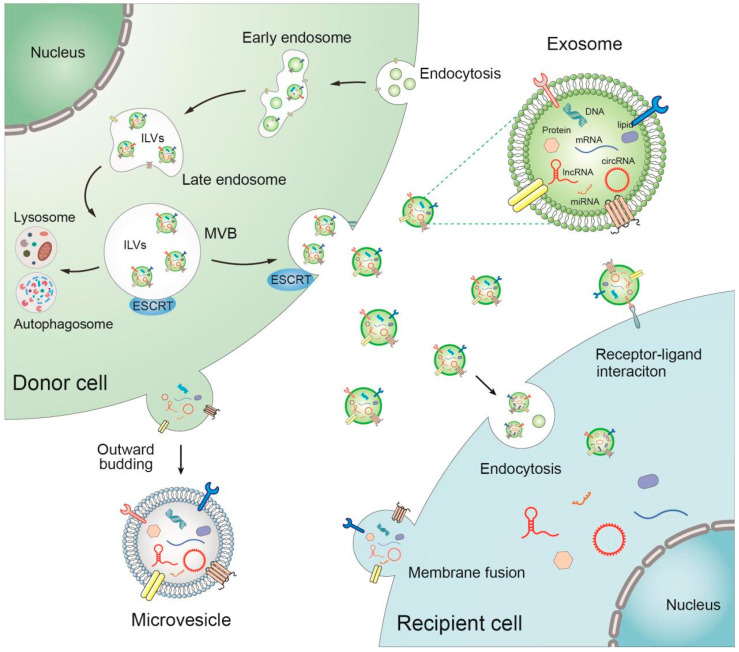
Biogenesis, release, and uptake of EVs. Exosome genesis begins with endocytosis on the surface of the cell membrane, with early exosomes forming by inward budding and subsequently maturing into late endosomes. MVBs either fuse with the plasma membrane to release exosomes into the extracellular environment or are degraded. MVs originate from direct outward budding and fission of the plasma membrane. Ultimately, the released EVs can be taken up by recipient cells through cellular endocytosis or membrane fusion, or display receptor–ligand binding.

EVs constitute a group of vesicles actively secreted by diverse cell types, playing a pivotal role in intercellular communication through the conveyance of intracellular substances. This section delineates the biogenesis, release, and uptake processes, with a primary focus on extensively investigated exosomes and MVs (Figure 1). MVs originate from direct outward budding and fission of the plasma membrane [39]. The genesis of exosomes commences with endocytosis at the cell membrane surface, where early exosomes are formed through inward budding. Early endosomes subsequently mature into late endosomes, encapsulating specific proteins, nucleic acids, and other substances to generate multiple luminal vesicles (ILVs), serving as the precursors of exosomes. Content sorting occurs at this time, involving two distinct mechanisms: the endosomal transit required sorting complex (ESCRT) pathway and an ESCRT-independent pathway. The main function of ESCRT is to sort specific components into ILVs. ESCRT is a complex system composed of four proteins, ESCRT-0, ESCRT-I, ESCRT-II, and ESCRT-III, each of which plays a different role in exosome biogenesis [40]. ESCRT-0 is responsible for cargo aggregation in the ubiquitin-dependent process. ESCRT-I and ESCRT-II act in tandem, allowing the endocytic membrane to encase specific molecules through germination. ESCRT-III drives vesicle division [41]. Alternatively, the oligodendroglial precursor cell line maintains an ESCRT-independent pathway that releases exosomes containing proteolipids [42]. The specific incorporation of ncRNAs into EVs undergoes regulation through diverse mechanisms, including the mediation of RNA binding proteins (RBPs), interaction with target RNAs, or post-transcriptional modification of ncRNAs. These processes actively participate in the selective sorting of specific lncRNAs and circRNAs into EVs [43,44,45,46]. Late nuclear endosomes contain multiple ILVs and develop into MVBs [21,27,47].

### 2.2. Release of EVs

MVBs undergo two distinct fates. Most MVBs are transported to lysosomes or autophagosomes for degradation, and a few MVBs contain CD63, LAMP1, and LAMP2 in their membranes, which mediate their fusion with the plasma membrane to release exosomes into the extracellular environment [27,41,48]. Reliance on the RAB family and the soluble N-ethylmaleimide-sensitive factor attachment protein receptor (SNARE) family is a recently elucidated feature of the exosome secretion process [49]. RABs are small GTPase proteins that control the germination, transport, and localization of intracellular vesicles on the plasma membrane, and RAB11 is the first RAB protein with reported association to exosome secretion [50]. A wide variety of RAB GTPases, known to switch from GDP-bound to GTP-bound states to activate effectors, participate in the regulation of MVB targeting. Rab7-dependent MVB transport to lysosomes has been well documented, and exosome secretion may be dependent on Rab7 ubiquitination status and endosomal cholesterol levels, regulating dynein movement-mediated MVB transport [51,52,53]. The culmination of exosome secretion is the fusion of MVBs with the plasma membrane, which is controlled by SNARE proteins and their regulators. SNARE, a complex of proteins that facilitates the fusion of two contacted plasma membranes, is instrumental in promoting vesicle membrane and plasma membrane fusion [54]. VAMP7, a member of the SNARE family, is indispensable for exosome secretion, given its capacity to regulate the fusion of MVBs with the plasma membrane [55]. In the context of tumor cells, exosome secretion relies on PKM2-mediated and H1HR-mediated phosphorylation of SNAP23. This phosphorylation event promotes the formation of SNARE complexes, facilitating the docking and fusion between MVBs and the plasma membrane, thereby facilitating exosome release [56,57]. However, MVs are released directly outside the cell by budding at the plasma membrane [39].

### 2.3. Uptake of EVs

EVs primarily transmit signals to recipient cells via direct contact with membrane protein ligands, thereby triggering molecular or phenotypic changes in recipient cells [58]. Alternatively, EVs modulate different biological processes in recipient cells by transferring their contents to target cells through cellular endocytosis or membrane fusion [59]. Exosomal cellular uptake systems include phagocytosis, lattice or fovea protein-mediated endocytosis, microcytosis, and lipid raft-mediated endocytosis, all of which are discrete systems allowing exosomes to enter the intracellular environment [60]. Various mediators participate in these interactions, including tetraspanins, extracellular matrix (ECM) proteins, integrins, proteoglycans, lectins, and lipids. Exosomal tetraspanins selectively recruit other membrane proteins, such as integrins, forming complexes that facilitate exosomal docking and uptake by target cells [61,62]. ECM proteins present on exosomes, such as fibronectin and laminin, interact with integrins on the cell surface, promoting cell docking and facilitating exosome uptake [63,64].

## 3. Commonalities in the Mechanisms of lncRNAs and circRNAs

LncRNAs represent transcripts exceeding 200 nucleotides in length, characterized by the absence of crucial open reading frames [65]. Operating as regulatory RNAs, lncRNAs exhibit selective packaging into exosomes [66]. LncRNAs, acting as messengers of intercellular communication, wield regulatory control over various aspects of tumor biology, including proliferation, invasion, migration, angiogenesis, drug resistance, and the remodeling of the tumor microenvironment (TME) [67,68,69,70]. Similarly, circRNAs constitute a subclass of non-coding RNAs, identified as pivotal bioactive molecules within tumor-derived EVs (TEVs), and are ubiquitous in all eukaryotic cells. CircRNAs have tissue-specific and cell-specific expression patterns, covalently block endogenous biomolecules, and their biogenesis is regulated by specific cis-acting elements and trans-acting factors [71]. Varied splicing processes result in three principal types of circRNAs: exonic circular RNAs, exonic intronic circular RNAs, and circular intronic RNAs [72]. CircRNAs function as regulators of gene and miRNA expression, modulating fundamental cancer processes like tumor cell proliferation, invasion, and metastasis, thereby contributing to cancer progression. Furthermore, circRNAs exhibit promise as diagnostic, prognostic, and predictive biomarkers [73].

**Figure 2:**
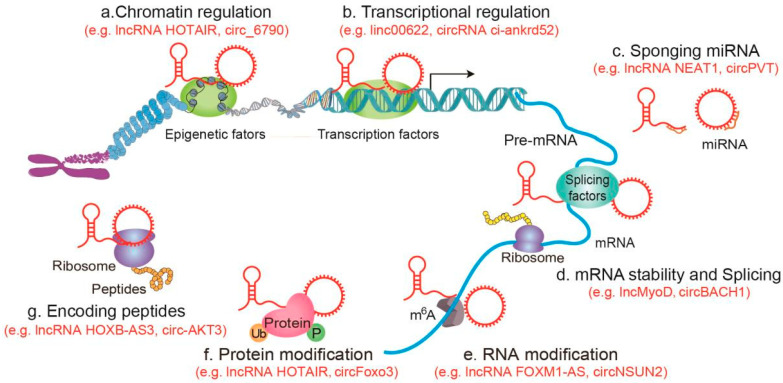
The commonalities in mechanisms of lncRNAs and circRNAs. Diverse mechanisms have been described for lncRNAs and circRNAs, including (**a**) chromatin regulation, (**b**) transcriptional regulation, (**c**) sponging miRNA, (**d**) mRNA stability and splicing, (**e**) RNA modification, (**f**) protein modification, (**g**) encoding peptides.

LncRNAs and circRNAs intricately influence cancer progression by orchestrating diverse levels of gene expression [33] (Figure 2). (a) Chromatin regulation: lncRNAs and circRNAs function at the epigenetic layer by recruiting multiple epigenetic factors to coordinate histone and DNA modifications [74,75,76]. For example, lncRNA HOTAIR scaffolds different histone modification complexes, participating in epigenetic regulation [74]. It has been found that circ_6790 regulates DNA methylation of S100A11 in pancreatic ductal adenocarcinoma (PDAC) by binding to chromosome box 7, ultimately inhibiting the progression of PDAC [77]. (b) Transcriptional regulation: lncRNAs and circRNAs regulate gene transcription by interacting with transcription factors, cofactors, or target gene promoters. For example, linc00622 can increase the expression of GABBR1 in neuroblastoma (NB) cells, inhibiting NB proliferation, invasion, and migration [78]. CircRNA ci-ankrd52 can form large aggregates at its transcription site, is associated with extended Pol II machinery, and displays a regulatory function as a positive regulator of Pol II transcription [79]. (c) Sponging miRNA: The competing endogenous RNA hypothesis (ceRNA) proposed by Pandolfi et al. in 2011 purports that endogenous RNA molecules contain miRNA action sites and competitively bind to miRNA, thereby indirectly regulating the effect of miRNA target genes [80]. The ceRNA hypothesis assigns new and broader biological functions to mRNAs and ncRNAs. The regulatory network composed of different types of RNAs, including miRNAs, lncRNAs, circRNAs, mRNAs, etc., plays an important role in biological processes through RNA-RNA interactions [81]. This has been confirmed in a variety of lncRNAs and circRNAs, such as lncRNA NEAT1, circPVT1 and circHIPK [82,83]. (d) mRNA stability and splicing: lncRNAs and circRNAs interact with RBPs, affecting protein translation by regulating mRNA stability and splicing. LncMyoD interacts with IGF2 mRNA binding protein 2, down-regulating the translation of mRNA involved in cell proliferation [84]. CircRNA circBACH1 binds to HuR protein and promotes translocation of HuR from the nucleus to the cytoplasm, thereby inhibiting translation of p27 protein and accelerating the development of hepatocellular carcinoma (HCC) [85]. (e) RNA modification: lncRNAs and circRNAs can participate in gene expression by mediating RNA modification. N6-methyladenosine (m6A) is the most prevalent mRNA internal chemical modification in eukaryotes. It has been found that the nuclear antisense lncRNA FOXM1-AS causes m6A methylation and promotes ALKBH5-FOXM1 interaction. Silencing ALKBH5 inhibits the proliferation of glioblastoma stem-like cells [86]. (f) Protein modifications: lncRNAs and circRNAs influence their activation and stability through protein modifications [86,87,88,89]. The lncRNA HOTAIR can serve as a protein-level control platform through the ubiquitin–proteasome pathway, and HOTAIR promotes the ubiquitination of Ataxin-1 by Dzip3 and Snurportin-1 via Mex3b, thereby accelerating their degradation in cells and in vitro [88]. CircFoxo3, p53, and E3 ubiquitin ligase MDM2 form a complex promoting MDM2-induced ubiquitination of p53, accelerating p53 degradation and inducing apoptosis [90]. (g) Encoding Functional Peptides: lncRNAs and circRNAs can encode functional peptides with pivotal roles in diverse biological processes [91,92]. LncRNA HOXB-AS3 encodes the highly conserved 53-aa peptide HOXB-AS3, which acts as a cancer suppressor [93]. Circ-AKT3 encodes the 174aa protein AKT3-174aa, inhibiting glioblastoma cell proliferation, radiation resistance, and tumorigenicity [94].

## 4. EV-lncRNAs and EV-circRNAs Act Between MSCs and Tumors

**Figure 3:**
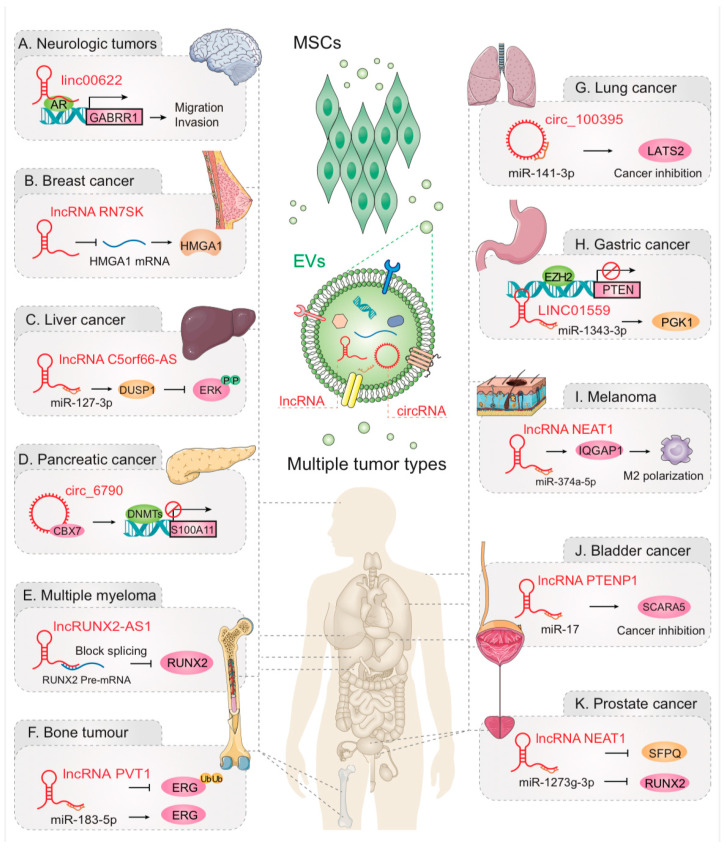
EVs regulate the relationship between MSCs and tumors by transferring different ncRNAs. The ncRNAs (lncRNAs and circRNAs) carried by different EVs are involved in the mutual communication between MSCs and various cancer cells (e.g., nervous system neoplasm, breast cancer, liver cancer, pancreatic cancer, multiple myeloma, bone tumor, lung cancer, gastric cancer, melanoma, prostate cancer and bladder cancer), thereby inhibiting or promoting cancer.

Currently, an escalating number of studies delve into the crosstalk between MSCs and tumors by EV-lncRNAs and EV-circRNAs, which are involved in a variety of cancer types. Here, we summarize the relevant studies reported so far in this field (Figure 3; Table 1).

### 4.1. Nervous System Neoplasm

NB is a common extracranial malignant tumor in children, which originates from embryonic neural crest cells and is the cause of 10% of tumor-related deaths in children [121]. Research indicates that EVs containing LINC00622 derived from adipose mesenchymal stem cells increase the expression of γ-aminobutyric acid type b receptor 1 (GABBR1) in NB cells by mediating the transcription factor androgen receptor to upregulate the activity of GABBR1, thereby inhibiting the proliferation, invasion, and migration of NB cells [78] (Figure 3A). Gliomas, which are neurological tumors, are among the most common lethal solid brain tumors and have a poor prognosis despite the efficacy of conventional treatments [122,123]. One study reported that exosomal lncRNA PTENP1, secreted by human umbilical cord MSCs, could be transferred into human glioma U87 cells and inhibit the cancerous progression of glioma cells through the PTENP1/miR-10a-5p/PTEN pathway. PTENP1 is reduced in cancer tissues compared to normal tissues, and lower PTENP1 levels predict worse overall survival, raising the possibility of PTENP1 as a novel diagnostic biomarker and treatment for glioma [95].

### 4.2. Breast Cancer

Triple-negative breast cancer (TNBC) is defined by the absence of estrogen receptor, progesterone receptor, and human epidermal growth factor receptor 2 expression. This subtype is characterized by a poor prognosis, exhibiting high rates of recurrence, metastasis, and mortality [124,125]. One study has shown that MSC derived exosomes deliver lncRNA RN7SK, which is overexpressed in TNBC cells, leads to the down-regulation of HMGA1 and its carcinogenic target genes in TNBC cells, and reduces the viability, proliferation, migration, invasion and epithelial mesenchymal transformation (EMT) of TNBC cells [96] (Figure 3B). Clinical data show that about 65–75% of patients with advanced breast cancer have bone metastases [126,127]. LncRNA SNHG3 is highly expressed in breast cancer cells. After SNHG3 knockdown, bone morphogenetic protein 3 is significantly down-regulated by miR-1273g-3p, resulting in bone lysolytic metastasis. SNHG3 knockdown inhibits the proliferation and migration of breast cancer cells [97]. These experimental findings support the therapeutic potential of targeting exosome-mediated crosstalk between cancer cells and bone marrow-derived MSCs (BMSCs) in the treatment of bone metastases.

### 4.3. Liver Cancer

HCC is the second leading cause of cancer-related death worldwide and is the most common liver cancer. Hepatitis B virus and hepatitis C virus infections are major risk factors [128,129]. Initial studies have shown that exosomes derived from cancer stem cells (CSCs) induce HCC tumor growth, progression, and metastasis, while exosomes derived from MSCs have the opposite effect [130]. As research continues to develop, studies have shown that lncRNA C5orf66-AS1, carried by MSC-derived exosomes, may inhibit the phosphorylation of ERK via enhancement of DUSP1 expression through miR-127-3p, and block the proliferation, migration, and invasion of liver CSCs through the miR-127-3p/DUSP1/ERK axis [98] (Figure 3C). Xu et al. report the overexpression of lncRNA FAM99B, carried by MSCS-derived exosomes, enhanced cell cycle arrest and apoptosis while inhibiting the viability, migration, and invasion of HCC cells [99].

### 4.4. Pancreatic Cancer (PC)

PC is a highly lethal malignancy with a 5-year survival rate of only 10% [131]. Yao et al. report that BMSC exosomal circ_0030167 enhanced Wif1 expression and inhibited Wnt8/β-catenin signaling pathway primarily through molecular sponge miR-338-5p. This factor inhibits the invasion, migration, proliferation, and stemness of PC cells, thereby inhibiting the progression of PC [100]. Wu et al. report that adipose-derived MSC (AMSC) derived EVs deliver NEAT1 to PC cells, NEAT1 regulates the expression of Snail and SOCS3 in PC cells by competitive binding to miR-491-5p, which promotes the proliferation, migration, and gemcitabine resistance of PC cells and enhances tumorigenicity in vivo [101].

Pancreatic Ductal Adenocarcinoma (PDAC) stands out as the predominant type of PC, renowned for its aggressive nature. With a median survival of less than 6 months and a 5-year survival rate below 5%, PDAC presents a formidable clinical challenge [132,133,134]. Gao et al. report that BMSC-derived exosomes transferred circ_6790 to PDAC cells to regulate S100A11 DNA methylation in PDAC by binding to chromosome box 7, thereby inhibiting PDAC cell invasion, migration, growth, and immune escape [77] (Figure 3D). This discovery sheds light on circ_6790 upregulation as a potential therapeutic avenue for PDAC. Previous studies have shown that tumor-derived exosome lnc-Sox2ot, a ceRNA of the miR-200 family, regulates Sox2, thereby inducing EMT, stem cell characteristics, and promoting PDAC invasion and metastasis. The level of lnc-Sox2ot in plasma exosomes is an independent risk factor for survival in PDAC patients [102]. This finding opens novel avenues for early detection, prevention, and therapeutic intervention in PDAC.

### 4.5. Multiple Myeloma (MM)

MM is characterized by the proliferation of clonal plasma cells into the bone marrow and the production of monoclonal immunoglobulin. Although treatment of MM has improved due to the discovery of novel therapeutics, the majority of MM patients still relapse [135,136,137,138,139]. Li et al. report that by transferring the MM-derived exosome lncRUNX2-AS1 to MSCs, RUNX2 and antisense lncRNA RUNX2AS1 transcripts hybridize and form RNA double strands through their base pair capabilities. This double-stranded transcription inhibits RUNX2 expression and participates in osteogenic inhibition by reducing splicing efficiency [103] (Figure 3E). LINC00461 is highly expressed in MM, and the abundance of LINC00461 is negatively correlated with the survival rate of MM patients. MSC-derived exosomes promote MM cell proliferation by regulating miR-15a, miR-16, and BCL-2 through LINC00461 [104]. Exosome-carried ncRNAs can be bidirectionally transferred between tumor cells and MSCs to regulate their respective functions.

### 4.6. Bone Tumor

Osteosarcoma (OS) stands as the most prevalent, primary malignant bone tumor globally, predominantly affecting children and adolescents [140,141]. Emerging, pivotal evidence demonstrates that exosomes derived from BMSCs package lncRNAs and circRNAs and transport them to OS cells in order to mediate the proliferation, migration, and invasion of OS cells. LncRNA PVT1 in BMSC-derived exosomes may promote the expression of ERG protein by sponge acting on miR-183-5p. Upregulation of PVT1 can also inhibit the ubiquitination and degradation of ERG, thereby maintaining the stability of ERG and ultimately promoting OS cell growth, proliferation, and migration [105] (Figure 3F). MSC-EVs, featuring lncRNA XIST and MALAT1, manipulate miR-655 and miR-143, respectively, to activate β-Catenin. This activation cascade propels OS cell proliferation, migration, and invasion, emphasizing the diverse roles of lncRNAs in OS progression [106,107]. Interestingly, two independent studies found that BMSC-EVs carried lncRNA NORAD into OS cells, which could promote tumor proliferation, invasion, migration, and inhibit angiogenesis by, respectively, regulating the miR-877-3p/CREBBP axis and the miR-30c-5p/KLF10 axis [108,109]. In addition to lncRNAs, circRNAs also play a role in OS. CircNRIP1 from MSCs, transported to OS cells via EVs, competes with miR-532-3p, thereby activating the PI3K/AKT signaling pathway. This mechanism enhances OS cell proliferation, migration, and invasion potential [110]. Ewing’s sarcoma (ES), another malignant bone tumor prevalent in children and adolescents, has attracted attention for its complex interactions with BMSC-EVs [142]. Huang et al. report that BMSC-EV-derived LINC00847 could inhibit the proliferation, migration, and invasion of ES cells and regulate their malignant phenotype [111].

### 4.7. Lung Cancer

Non-small cell lung cancer (NSCLC) is a predominant contributor to cancer-related mortality, constituting approximately 85% of all lung cancer cases, with a survival rate of less than 15% [143,144]. AMSCs over-express circ_100395, inhibit the activity of the Hippo signaling pathway through sponge miR-141-3p, increase the expression of LATS2, and inhibit the growth of NSCLC in vivo [112] (Figure 3G). This study suggests that overexpression of circ_100395 in AMSC-derived exosomes is a new approach to the treatment of NSCLC. Furthermore, the bidirectional communication between MSCs and cancer cells, facilitated by tumor-derived exosomes, has been identified. For instance, exosomes derived from lung tumor cell A549 possess the ability to impede osteogenesis and adipose differentiation of AMSCs. This process, in turn, promotes the development of lung tumors, highlighting the dynamic and reciprocal nature of MSC-cancer cell interactions in the TME [145].

### 4.8. Gastric Cancer

Gastric cancer is the fourth most frequently diagnosed cancer and the third leading cause of cancer death worldwide. Annually, it contributes to over 1 million new cases and nearly 769,000 deaths [146]. Wang et al. report MSC-derived exosome LINC01559 activates the PI3K/AKT pathway through sponge miR-1343-3p to up-regulate PGK1 and recruit EZH2 to inhibit PTEN, which promotes the proliferation, migration and stemness of gastric cancer cells [113] (Figure 3H). Similarly, circRNAs have been studied in gastric cancer. For example, gastric adenocarcinoma cell-derived exosomes package circ_0004303 and transport it to MSCs, where circ_0004303 through sponge miR-148a-3P. Upregulation of activated leukocyte cell adhesion molecule level regulates the migration and invasion of human AMSCs [114]. This study indicates that tumor cells recruit MSCs to adjacent tissues through exosome secretion. Additionally, overexpression of gastric cancer stem cell-derived exosome circ670 significantly promoted the stem cell character and EMT of gastric CSCs [115]. All these studies may provide new targets for the treatment of gastric cancer.

### 4.9. Other Tumors

Melanoma is a complex and genomically diverse malignant tumor, and melanoma derived from melanocytes is the most aggressive skin cancer [147,148]. The lncRNA NEAT1, derived from BMSC-EVs, promotes M2 macrophage polarization through the miR-374a-5p/LGR4/IQGAP1 axis, thereby promoting tumor formation and angiogenesis in melanoma [116] (Figure 3I). Studies on lncRNAs have also been reported in genitourinary tumors. Prostate cancer is the most common cancer in men and the second leading cause of cancer-related death, and up to 90% of patients with advanced prostate cancer will develop bone metastases [149,150]. It has been shown that prostate cancer-derived exosomes enhance NEAT1 expression in BMSCs. Secondly, NEAT1 upregulates dwarf-associated transcription factor 2 (RUNX2) expression through competitive binding to miR-205-5p. Lastly, NEAT1 can be facilitated by the SFPQ/PTBP2 axis expression of RUNX2. This process ultimately promotes the osteogenic differentiation potential of BMSCs and induces osteogenesis in vivo [117] (Figure 3J). Bladder cancer, the ninth most common urogenital cancer globally, exhibits a higher incidence in men [151,152]. BMSC-derived exosomes can transport lncRNA PTENP1 into bladder cancer cells, and lncRNA PTENP1 inhibits the malignant phenotype of bladder cancer cells by regulating the miR-17/SCARA5 axis [118] (Figure 3K). Studies have shown that the MSC-derived exosome circ_0037104 enhances the expression of APAF1 by regulating miR-620, thereby inhibiting the proliferation and metastasis of cholangiocarcinoma cells [119]. The exosomal lnc RNA SNHG5 produced by acute myeloid leukemia (AML) cells leads to chemotherapy resistance of AML cells through interaction with polypyrimidine tract-binding protein 1 [120].

## 5. Clinical Application of EVs

**Figure 4:**
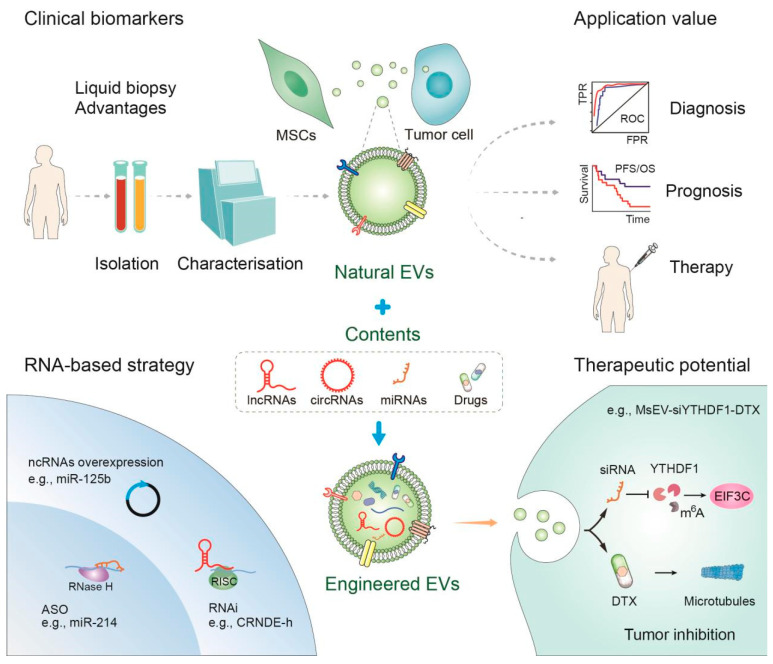
Clinical application value of EVs. The role of EVs in clinical diagnosis, prognosis, and treatment, and the therapeutic potential of engineered EVs.

EVs, being vesicles naturally secreted by cells, play a crucial role in intercellular information transmission and are implicated in the onset and progression of various diseases, serving as disease biomarkers (Figure 4). The research on EV-derived lncRNAs and circRNAs as a non-invasive biomarker is advancing rapidly and is expected to become a real-time monitoring tool for the dynamic changes in tumors. In the research on solid tumors, some of the EV-derived lncRNAs and circRNAs can also serve as biomarkers for predicting treatment response and prognosis [153]. For instance, in the distinction between HCC and healthy controls, the ROC curve analysis results of lnc RNA-RP11-513I15.6 derived from EVs demonstrated satisfactory sensitivity and specificity [154]. Notably, MSCs exhibit a superior exosome secretion capacity compared to other cell types [155]. Furthermore, MSCs derived from diverse human tissues have obtained regulatory approval for clinical applications in several countries [156]. MSC-EVs may act as a paracrine mediator, which can regulate the proliferation, metastasis, and angiogenesis of tumor cells through the transfer of signaling molecules [157,158]. Since MSC-EVs have the same functions as their parent cells in tissue repair and regeneration, it is considered an alternative to MSC-based treatments [159,160,161]. With characteristics amalgamating the advantages of EVs and MSCs, MSC-EVs are progressively employed as nanodrug delivery carriers [162].

MSC-EVs have many advantages: (i) MSC-EVs are a naturally occurring endogenous carrier with high biocompatibility and low immunogenicity, allowing them to avoid activation of immune responses and thus avoid clearance by the human immune system [163,164]. At the same time, the immune evasion property of MSC-EVs makes it easier to administer repeatedly [165]. (ii) Due to its small size, MSC-EVs have strong permeability and retention properties in solid tumors. The permeability allows it to freely cross biological barriers such as the blood-retinal barrier and the blood–brain barrier, and the retention properties enable it to target and aggregate at the disease site, which has a strong tumor targeting ability [166,167]. (iii) An additional significant advantage lies in the intrinsic tumor affinity inherited from their parental cells. The complex surface proteins of MSC-EVs offer engineering opportunities for the incorporation of exogenous targeting ligands and other surface modification strategies, thereby enhancing their targeting capabilities [168]. (iv) In addition, the solid lipid bilayer structure of MSC-EVs protects its loaded contents from harsh TME and avoids cytotoxicity of the cellular phagocyte–lysosomal pathway [169]. (v) In terms of pharmacokinetics, MSC-EVs are able to deliver their cargo with minimal immune clearance and excellent systemic retention in vivo [58,170]. MSC-EVs have been used as a drug carrier for the treatment of a variety of diseases, including tumors, and have achieved satisfactory results [171].

In fact, the function of MSC-EVs mainly depends on the substances they carry, and MSC-EVs are currently used in the diagnosis and treatment of a variety of diseases [172] (Figure 4). A clinical study conducted at Cairo University in Egypt explored the important role of miR-136, miR-494, and miR-495 in the diagnosis of eclampsia by MSC-derived exosomes [173]. On the therapeutic front, an impactful prospective non-randomized open-label cohort study, initiated in April 2020, substantiated the safety and efficacy of exosomes derived from allogeneic BM-MSCs in treating 24 patients afflicted with severe COVID-19 [174]. Another notable clinical trial, presently in phase 1 (NCT03608631), investigated the optimal dose and side effects of mesenchymal stromal cell-derived KrasG12D siRNA exosomes in patients with KRASG12D-mutated pancreatic cancer that had spread to other body sites.

Certain ncRNAs exhibit potential to impede tumor progression, highlighting promising treatment strategies for cancer. Notably, the immune activity of EV-derived miRNAs such as miR-125b and miR-34a has emerged as a promising avenue for cancer treatment [175,176]. The exosome miR-199a, derived from MSCs, was shown to increase chemotherapy sensitivity of HCC cells via the mTOR pathway [177]. Additionally, MSCs-derived exosomal miR-122 significantly augments the anti-tumor efficacy of sorafenib in vivo by facilitating communication between MSCs and HCC cells [178]. Conversely, the suppression of certain ncRNAs has been shown to impede cancer progression. For instance, circRNA-002178, which is significantly upregulated in exosomes within the serum of lung adenocarcinoma patients, exhibits suppressive effects on cancer [179]. Tumor-secreted miR-214 induces Treg-mediated immunosuppression, a process mitigated by delivering functional anti-miR-214 antisense oligonucleotides via MVs to CD4 + T cells [180]. Additionally, the silencing of lncRNA CRNDE-h through shRNA targeting attenuates colorectal tumor growth in mice [181].

Furthermore, the synergistic application of EVs with engineering techniques has demonstrated the potential to enhance drug targeting to tumors, elevating the local concentration of EVs in strategic locations. This approach not only amplifies therapeutic efficacy but also mitigates unnecessary systemic toxicity [182,183]. At present, an increasing number of research directions involve integrating electric vehicles with engineering technology, significantly enhancing their targeted capabilities and providing crucial technical support for clinical applications. The targeted modification of EVs can evade the host immune system and achieve precise targeted drug delivery. The current targeted modification of EVs can be classified into biological modification, physical modification, and chemical modification. Biological modification is a widely used membrane modification method. The most common ones include targeting peptides and aptamers, etc. Modifying EVs with targeting peptides can significantly enhance their targeting ability for tissues and organs, and improve the cumulative effect of chemotherapy drugs, etc. [184]. The connection of the aptamer to the surface molecules of exosomes is an emerging method for surface functionalization of EVs. It enables interaction with specific ligands and can target tumor cells with high affinity and specificity [185]. Engineering EVs can achieve more accurate tumor targeting under external physical conditions, such as electrostatic, magnetic, ultrasound, and laser irradiation [186]. The use of acidic tumor microenvironment in chemical modification for achieving controlled drug release represents a highly promising approach for developing efficient drug delivery systems tailored to different clinical stages [187]. In a separate study, the therapeutic efficacy of engineered MSC-EVs enriched with miR-302a was explored in endometrial cancer (EC). By suppressing the expression of cyclin D1 and inhibiting the AKT signaling pathway, MiR-302a demonstrated the capacity to inhibit EC cell proliferation and migration [188]. Another innovative strategy involved the loading of doxorubicin into MSC-derived exosomes through electroporation, resulting in the inhibition of colon cancer growth. Notably, tumor accumulation was significantly higher with doxorubicin-loaded exosomes compared to free doxorubicin [189]. Engineered EVs improve some shortcomings of natural EVs and expand their advantages, making them more efficient, safe, and more suitable for clinical use. It is expected to become an efficacious drug delivery vector and a promising treatment strategy.

## 6. Conclusions

This review presents a comprehensive overview of the intrinsic characteristics of MSCs and EVs, encompassing the biogenesis, uptake, and release dynamics of EVs, along with the prevalence of molecular mechanisms of lncRNAs and circRNAs. Furthermore, it provides insights into the involvement of lncRNAs and circRNAs conveyed by MSC-EVs or TEVs in diverse cancer types. Lastly, the review explores the potential clinical applications of MSC-EVs and highlights the advantages and prospects of engineered EVs.

In recent years, the research on the role of ncRNAs in tumors has introduced novel perspectives for the diagnosis and treatment of tumor diseases. MSC-EVs, serving as an ideal drug delivery vehicle, have been used in the treatment of numerous diseases. However, several limitations remain unaddressed. (i) Current separation technologies lack absolute purification capability, each possessing distinct advantages and limitations. The International Extracellular Vesicle Society (ISEV) recommends a combination of separation techniques for optimal EV enrichment, emphasizing the necessity for thorough EV characterization according to ISEV guidelines to validate isolation efficacy [190]. (ii) A rapid and cost-effective technique for large-scale EV isolation remains elusive, hindering widespread clinical application. Existing research on EVs primarily operates at the microgram level, insufficient for potential clinical requirements [191]. The quality control and determination of dosage are also difficult. (iii) Some MSC-EVs can promote tumor progression, highlighting the need to carefully characterize EV contents. However, the detailed characterization of the subpopulations of EVs and the molecular composition of each EV type remains unknown [192]. Future studies should distinguish between different MSC-EV subsets and further explore their roles and related mechanisms in cancer development. (iv) In addition, the route of administration, dose, efficiency, and cost of EVs as drug carriers require comprehensive evaluation and improvement. An in vivo monitoring platform is also needed to optimize drug distribution to ensure treatment safety [193]. (v) Considering the complexity and heterogeneity of MSC-EV functions, if they are to be applied in a large, clinic-wide scale, comprehensive systematic standards, encompassing culture conditions, modification, production, purification, characterization, and storage, are essential. Long-term safety and therapeutic efficacy of MSC-EVs need to be further verified. (vi) Although MSC-EVs are relatively ideal drug delivery systems, the targeting efficiency still demands further improvement. Currently, targeting can be enhanced by engineering methods, but this is operationally complex and requires further experimental exploration [182]. In conclusion, the MSC-EVs drug delivery system holds significant promise for targeted tumor therapy. As bioengineering strategies for EVs progress rapidly, emerging technologies are expected to enhance the anticancer drug delivery capabilities of MSC-EVs, ultimately benefiting a broader spectrum of cancer patients in the future.

## Figures and Tables

**Table 1:** The emerging roles of EV-ncRNAs between MSCs and different cancer types.

ncRNA	Source Cells	Target Cells	EVs Types	Expression	Function	Mechanism	Refs
circ_6790	MSCs	PDAC	Exosomes	Up	Cancer inhibition	Chromatin regulation	[77]
LINC00622	MSCs	Neuroblastoma	EVs	Up	Cancer inhibition	Transcriptional regulation	[78]
lncRNA PTENP1	MSCs	Gliomas	Exosomes	Down	Cancer inhibition	Sponging miRNA	[95]
LncRNA RN7SK	MSCs	TNBC	Exosomes	Up	Cancer inhibition	Transcriptional regulation	[96]
lncRNA-SNHG3	MSCs	Breast cancer	Exosomes	Up	Cancer promotion	Sponging miRNA	[97]
lncRNA C5orf66	MSCs	HCC	Exosomes	Up	Cancer inhibition	Sponging miRNA	[98]
lncRNA FAM99B	MSCs	HCC	Exosomes	Down	Cancer inhibition	—	[99]
circ_0030167	MSCs	PC	Exosomes	Up	Cancer inhibition	Sponging miRNA	[100]
lncRNA NEAT1	MSCs	PC	EVs	Up	Cancer promotion	Sponging miRNA	[101]
lncRNA-Sox2ot	Cancer cells	PDAC	Exosomes	Up	Cancer promotion	Sponging miRNA	[102]
lncRUNX2-AS1	Cancer cells	MSCs	Exosomes	Up	Cancer inhibition	Transcriptional regulation	[103]
LINC00461	MSCs	MM	Exosomes	Up	Cancer promotion	Sponging miRNA	[104]
lncRNA PVT1	MSCs	Osteosarcoma	Exosomes	Up	Cancer promotion	Sponging miRNA	[105]
lncRNA XIST	MSCs	Osteosarcoma	Exosomes	Up	Cancer promotion	Sponging miRNA	[106]
lncRNA MALAT1	MSCs	Osteosarcoma	EVs	Up	Cancer promotion	Sponging miRNA	[107]
lncRNA NORAD	MSCs	Osteosarcoma	EVs	Up	Cancer promotion	Sponging miRNA	[108]
lncRNA NORAD	MSCs	Osteosarcoma	Exosomes	Up	Cancer promotion	Sponging miRNA	[109]
circNRIP1	MSCs	Osteosarcoma	EVs	Up	Cancer promotion	Sponging miRNA	[110]
LINC00847	MSCs	Ewing’s sarcoma	EVs	Down	Cancer inhibition	Sponging miRNA	[111]
CircRNA_100395	MSCs	Lung cancer	Exosomes	Down	Cancer inhibition	Sponging miRNA	[112]
LINC01559	MSCs	Gastric cancer	Exosomes	Up	Cancer promotion	Sponging miRNA	[113]
Circ_00004303	Cancer cells	MSCs	Exosomes	Up	Cancer promotion	Sponging miRNA	[114]
circ670	Cancer stem cells	Gastric cancer	Exosomes	Up	Cancer promotion	—	[115]
lncRNA NEAT1	MSCs	Melanoma	EVs	Up	Cancer promotion	Sponging miRNA	[116]
LncRNA NEAT1	Cancer cells	MSCs	EVs	Up	Cancer promotion	Sponging miRNA	[117]
lncRNA PTENP1	MSCs	Bladder cancer	Exosomes	Up	Cancer inhibition	Sponging miRNA	[118]
circ_0037104	MSCs	Cholangiocarcinoma	Exosomes	Down	Cancer inhibition	Sponging miRNA	[119]
lncRNA SNHG5	Cancer cells	Acute myeloid leukemia	Exosomes	Up	Cancer promotion	mRNA stability	[120]

Abbreviations: TNBC: triple negative breast cancer; HCC: hepatocellular carcinoma; MSCs: mesenchymal stem cells; PC: pancreatic cancer; MM: multiple myeloma; PDAC: pancreatic ductal adenocarcinoma.
